# Explaining mobile government social media continuance from the valence perspective: A SEM-NN approach

**DOI:** 10.1371/journal.pone.0246483

**Published:** 2021-02-04

**Authors:** Yubo Peng, LingWu Wang, Shuiqing Yang

**Affiliations:** 1 Department of E-Commerce Operation, Zhejiang Global E-commerce Research Institute, Hangzhou, Zhejiang, People’s Republic of China; 2 School of Information Management and Engineering, Zhejiang University of Finance and Economics, Hangzhou, Zhejiang, People’s Republic of China; Shandong University of Science and Technology, CHINA

## Abstract

Different from many previous studies explain mobile social media usage from a technical-center perspective, the present study investigates the factors that influence citizens’ mobile government social media (GSM) continuance based on the valence framework. The research model was calculated by using data collected from 509 citizens who are the mobile GSM users in China. A structural equation modeling (SEM)-neural network (NN) method was employed to test the research model. The results of SEM indicated that the positive utilities included social value and hedonic value positively affect mobile GSM continuance, while the negative utility reflected by self-censorship negative affect mobile GSM continuance. This is further supported by the results of the neural network model analysis which indicated that hedonic value is more influencing predictor of continuous usage of mobile GSM, following by social value and self-censorship.

## Introduction

Due to the nature of its social interaction, mobile social media has gained popularity among citizens in the past few years [1–3]. According to the 45th CNNIC, the total number of government social media (GSM) users in China has exceeded 694 million by March 2020, accounting for 76.8% of the total Internet users [4]. In the meantime, the number of the official approved Chinese government microblog accounts has already reached 138, 854 [4]. However, despite it has been widely accepted by many citizens, its continuous use rate is extremely low [5]. For example, although the Guangzhou government’s GSM “Guangzhou Announcement” has gained over 5.1 million followers [6], the number of sharing of the GSM topics is still very low. How to lead citizens to continue using of mobile GSM is an important question that needs to be answered.

Extant studies tend to explored the mobile GSM usage behaviors based on the leading TAM (technology acceptance model) and its extensions [7, 8]. Some researchers argued that these technology performance-based theories cannot explain “why and how” people decide to use a specific social media to satisfy their needs [9]. A review of existing literature shows that the valence framework may offer an explanation why citizens decide to continue using the mobile GSM [10]. The valence framework proposed that people make a particular usage decision based on their beliefs to maximize expected positive utilities and to minimize expected negative utilities [10, 11]. Therefore, the present study intends to apply the valence framework in the mobile social media context to explain citizens’ mobile GSM continuance behaviors. In addition, despite many studies have examined the factors that affect mobile GSM behaviors, most of the research has focused on the benefits of mobile GSM usage [5, 9], little research has explored the impacts of self-censorship on citizens’ benefits or utilities evaluation and consequent mobile GSM continuance behaviors. In fact, mobile GSM not only can promote personalized microblog to citizens based on their preferences and location, but also can allow citizens to receive and share messages within the microblogging platform instantly. Despite mobile GSM brings much benefits to citizens, it also may induce citizens’ negative utilities (e.g., privacy concerns and self-censorship) during their mobile GSM interactions. The underly mechanism of how citizens’ both positive and negative utilities evaluations may affect their continuance behaviors in the context of mobile GSM is still unknown. Therefore, it is important to explore the influences of citizens’ positive and negative utilities on mobile GSM usage behaviors.

Based on the valence framework, the present study intends to explore the two questions: (1) How does citizens’ positive (e.g., social value and hedonic value) and negative (e.g., privacy concerns and self-censorship) utilities affect their mobile GSM continuance intention? (2) What are the roles of citizens’ positive and negative utilities on forming their mobile GSM continuance intention?

The contributions of our study are threefold: first, unlike many previous studies explain mobile GSM behavior by using the leading technology acceptance model and its expansion, the present study applied the valence framework in the mobile GSM context and examined the influences of citizens’ positive and negative utilities on mobile GSM usage. Second, unlike extant studies usually focused on the benefits of mobile GSM usage, the present study explored the impacts of citizens’ both the benefits and cost assessment on their usage behaviors. Finally, unlike many previous studies tend to employ the linear SEM models to explain user behaviors, the present study adopts a SEM-NN combined method to explain the determinants of mobile GSM continuance. The SEM-NN combined method can take the advantages of two approaches (The SEM and neural network modeling).

## Literature review

Nowadays, government microblog services in China are utilized by the government agencies and officials as the most important social media tool to disclose authoritative information, and encourage public opinion expression [1, 5]. Especially in recent years, fueled by the pervasiveness of mobile devices and wireless Internet, mobile GSM opens up new opportunities by allowing citizens to share comments, suggestions and other feedback with government and other citizens without the limitation of time and location [5].

In recent years, mobile GSM usage has been examined by many scholars [1, 12]. For instance, Ahmad and Khalid [12] employed TAM theory to explore the factors that influence mobile government services adoption from the user’s perspective. They found that trust and social influence affect mobile government services usage intention. Based on the unified theory of Acceptance and Use of Technology model, Sharma, Al-Badi [7] examined the factors that influence mobile government services (MG-App) usage intention, and found that trust and performance expectancy exerts strong impacts on citizens’ MG-App acceptance. Many studies examined the post-adoption or continuance usage of mobile GSM [1, 12]. For instance, Yang, Hui [9] found that perceived citizen-government interactivity and perceived integration influence both citizens’ extrinsic and intrinsic value, which further affect mobile government microblogging continuance. More recently, Li, Wang [1] conceptualized perceived interactivity as a second-order construct which included playfulness, control, responsiveness, and connectedness, and explored its impacts on citizens’ government microblogging continuance. They found that perceived playfulness, control, and connectedness affect citizens’ trust and satisfaction, which further influence their government microblogging continuance.

Despite many previous studies have examined mobile GSM continuance behaviors [1, 5, 9], rare of studies has explored citizens’ continuance intention by considering the impacts from both positive and negative utilities. What is more, the existing studies mainly centered on the mobile GSM usage’s benefits aspects, rare of study has examined the impacts of self-censorship on citizens’ continuance intention of mobile GSM. Indeed, disclosure in publicly available microblogging platforms may cause citizens to worry about the potential risks related to such information disclosure. For instance, the privacy concerns may ultimately hinder continuance usage of the mobile GSM, which will further hinder the long-term growth of the government microblogging. Therefore, based on the valence framework, the present study aims to explore the impacts of citizens’ positive and negative utilities on their mobile GSM continuance.

## Theoretical background and research hypotheses

**Fig 1:**
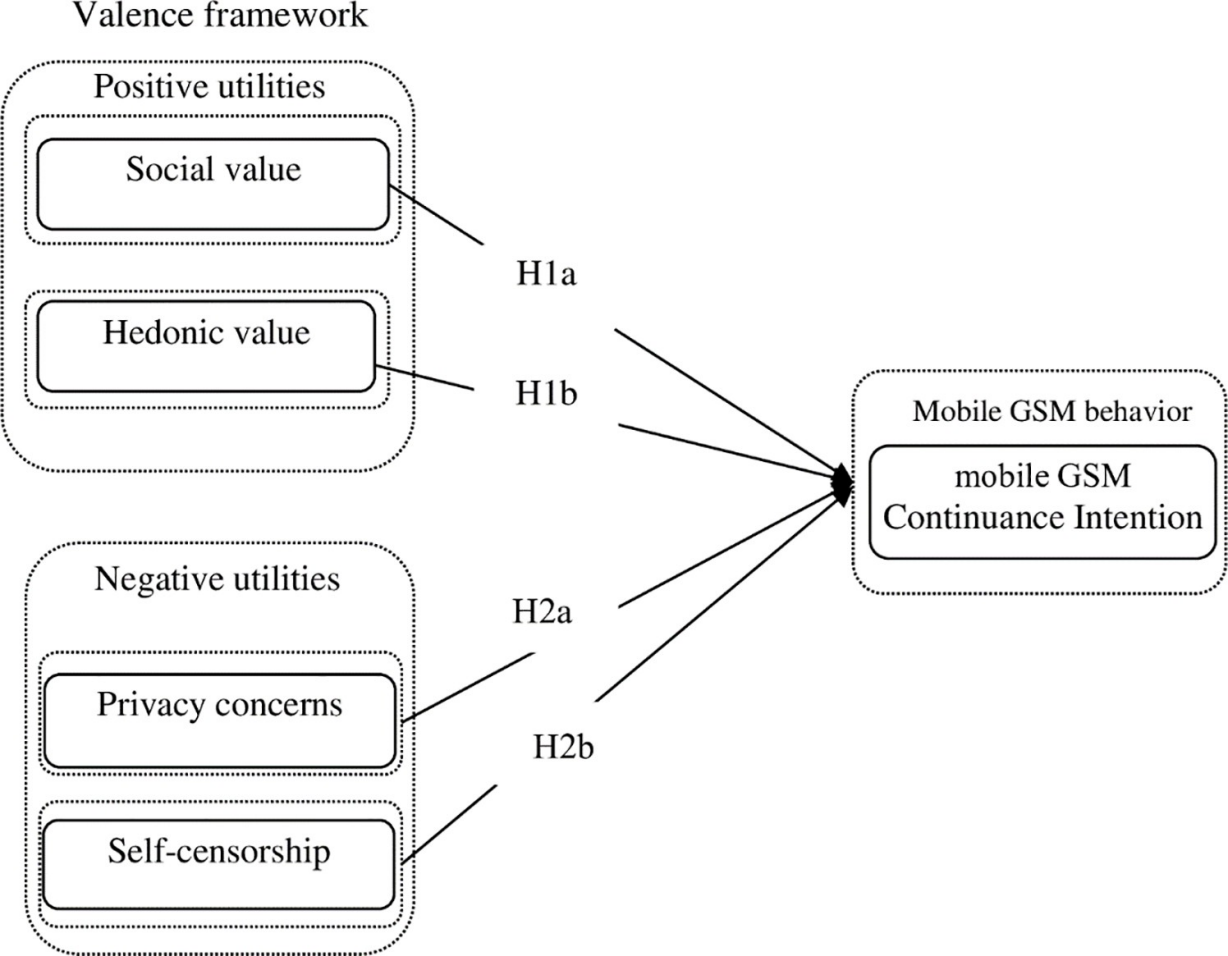
Research model.

Based on the valence framework, a research model reflects citizens’ positive and negative utilities, and their impacts on subsequent mobile GSM continuance was developed. The proposed research model is shown in Fig 1.

### The valence framework

Originally developed from the economics and psychology literature, the valence framework explained individuals’ behaviors that incorporated both the positive and negative utilities of their decision [10]. According to the valence framework, people act to minimize expected negative utility and maximize expected positive utility associated with a particular decision-making behavior [10, 11].

The valence framework has been applied in various research environments including online shopping [13], mobile payment [11], and sharing economy [14]. For instance, Kim, Ferrin [13] applied the valence framework in the context of online shopping, and found that perceived benefits as the positive utility positively affects willingness to purchase, while perceived risk as the negative utility negatively influences willingness to purchase. Yang, Lu [11] extended the valence framework to the mobile Internet environment to explain mobile payment continuance. They found that compatibility and relative advantage affect continuance intention, while perceived risk negatively influences continuance intention. More recently, Lee, Chan [14] found that perceived benefits, perceived risks, trust in the platform significantly affect Uber users participate intention in the sharing economy context. Based on the extant studies, the present research applied the valence framework in the mobile social network context to explore the impacts of both positive and negative utilities on mobile GSM continuance.

### The positive utilities

According to the original valence framework, perceived benefits reflects the positive utility of users’ decision to use a particular technology. In the context of the present study, we should capture the positive unities based on the mobile GSM features, such as social value and hedonic value. Social value has long been regarded as the important factor that affects social media usage [9]. In the present study, when citizens can easily interact with government agencies or other citizens via the mobile GSM platform, they will be more likely to develop positive utility evaluations on the mobile GSM. Previous studies also found that social value for maintaining social relationships positively affect users’ continuance intention [9]. Therefore, we can propose the following hypothesis:

H1a: Social value will positively affect citizens’ mobile GSM continuance intention.

The impacts of hedonic value on citizens’ continuance intention have also validated by the extant studies [5, 15]. For instance, Ding, shuiqing [5] examined the impacts of citizens’ gratifications on mobile government social media participation behaviors. They found that hedonic value has positive impact on participation behaviors. In the present study, it is expected that citizens will tend to continue using mobile GSM if they have experienced a higher hedonic value. Therefore, following on the extant studies [5, 15], we can propose the following hypothesis:

H1b: Hedonic value will positively affect citizens’ mobile GSM continuance intention.

### The negative utility

When using mobile GSM, citizens have to bear the privacy concerns related to their usage decision [16]. In the original valence framework, the negative utility is mainly reflected by risk associated with the usage decision. In the context of mobile government social media, privacy concerns occurred when citizens using mobile GSM services, which was not considered in the original valence framework. The significant negative impacts of privacy concerns on usage behaviors have been validated in the various environment including virtual health community’s usage [17], smart services resistance [18], and retail technologies usage [19]. Therefore, based on the extant studies [17, 19], we can propose the following hypothesis:

H2a: Privacy concerns will negatively affect citizens’ mobile GSM continuance intention.

In the present study, self-censorship will also have negative impact on mobile GSM continuance intention. Self-censorship refers to an individual suppresses her or his own attitudes where a pubic censor is either irrelevant or absent [20]. In the social media environment, self-censorship reflected the fact that users filter their own thoughts or expression before shared them to the social media platform [21]. In the context of the present study, when citizens experience a high level of self-censorship, they will be more likely to discontinue use of mobile GSM. Therefore, based on the extant studies [20, 21], we can propose that:

H2b: Self-censorship will negatively affect citizens’ mobile GSM continuance intention.

## Methodology

### Instrument

All constructs in the present study were borrowed from the existing well-established studies to ensure content validity. The items of privacy concern were adapted from James, Wallace [16]. Three items of self-censorship were adapted from Ranzini and Hoek [22]. The items of social value were borrowed from Zhang, Li [23] and Guo, Liu [24]. Three items of hedonic value were adapted from Zhang, Li [23]. The items of continuance intention were borrowed from Bhattacherjee [25]. A seven-point Likert scale was used to measure the instrument, with response choices ranging from (1 = strongly disagree) to (7 = strongly agree). A back-translation procedure was performed to ensure the translation validity from the original English items to the Chinese scales. A pilot test was also conducted on 36 mobile GSM users to further examine the readability and clarity of the instrument. The final questionnaire is displayed in the S1 Table.

### Data collection

The present study conducted an online survey on a well-known web-based survey platform (WJX.CN) to collect empirical data. The sample pool of WJX.CN has 2.6 million registered members from different cities. The sample services offered by this online survey site were adopted for our sample collection. Due to the objective of our study is to investigate citizens’ mobile GSM usage behaviors, the participants should have certain usage experience of mobile GSM. Therefore, the filter conditions in our survey project are limited to those who had mobile microblogging services usage experience in Sina microblog. WJX.CN then randomly invited eligible respondents to participate in the web-based survey. Participants in this study have been properly instructed and are voluntarily participating in our survey. Therefore, the participant’s consent has been obtained. In addition, the data were analyzed anonymously. The data collection process lasted for three weeks (from September 20, 2019 to October 12, 2019). All responses were scrutinized and discarded whose who given the same answer for all questions, and whose who did not have usage experiences of mobile GSM. Finally, 509 valid response were obtained. The demographic statistics of the sample are listed in Table 1.

**Table 1:** Sample demographics.

Measure	Item	Number(N = 509)	percentage (%)
Gender	Male	253	49.7%
	Female	256	50.3%
Age	<18 years	3	0.6%
	18~25 years	78	15.9%
	26~35 years	320	62.9%
	>35 years	108	21.2%
Education level	High school or below	12	2.4%
	Two-year college	47	9.2%
	Four-year college	407	80%
	Graduate school or above	43	8.4%
Occupation	Student	22	4.3%
	General Employee	308	60.5%
	Manager	154	30.3%
	Self-employed	13	2.6%
	Others	12	2.4%
Mobile GSM experience	<1 year	45	8.9%
	1~3years	246	48.3%
	3~5 years	162	31.8%
	>5 years	56	11%
Main followed GSM	Hangzhou Releases	97	19.1%
	Shanghai Releases	117	23%
	Beijing Releases	177	34.8%
	Guangzhou Releases	65	12.8%
	Others	53	10.3%

## Data analysis and results

### Reliability and validity

The reliability and validity of the scales were tested by conducted a confirmatory factor analysis. As shown in Table 2, for each construct, the coefficient of Cronbach’s alpha and composite reliability (CR) were all higher than the recommended value of 0.7, showing good reliability of the constructs. The loadings of the items are all above the recommended level of 0.7, and the constructs’ average variance extracted (AVE) values were all over the recommended benchmark of 0.5, indicating good convergent validity of the constructs.

**Table 2:** Scale properties.

Variable	Item	Standard Loading	Cronbach’s Alpha	CR	AVE
Social value (**SOV**)	SOV1	0.805	0.810	0.887	0.725
	SOV2	0.883			
	SOV3	0.863			
Hedonic value **(HEV)**	HEV1	0.866	0.827	0.896	0.742
	HEV2	0.852			
	HEV3	0.865			
Privacy concerns **(PCO)**	PCO1	0.850	0.883	0.928	0.811
	PCO2	0.939			
	PCO3	0.909			
Self-censorship **(SCE)**	SCE1	0.861	0.794	0.877	0.705
	SCE2	0.767			
	SCE3	0.885			
Continuance Intention **(CON)**	CON1	0.842	0.824	0.895	0.740
	CON2	0.844			
	CON3	0.852			

Note: PCO = Privacy concerns; SCE = self-censorship; SOV = social value; HEV = hedonic value; CON = Continuance intention.

The discriminant validity was then tested by compared each construct’s inter-construct correlation and its square root of the AVEs. Following the procedure conducted by [26], we adopted both Fornell-larcker Criterion and Heterotrait-Monotrait Ratio (HTMT) for testing discriminant validity. As displayed in Table 3, for all constructs, the square root of the AVEs were significantly higher than their corresponding inter-construct correlation coefficients, indicting good discriminant validity [27]. To further assess the validity of construct, the cross-loading matrix was listed in S2 Table. The internal-construct loading of each extracted factor was higher than its cross-loading on other factors, displaying a clear loading matrix.

**Table 3:** Fornell-larcker criterion and Heterotrait-Monotrait Ratio (HTMT).

		SOV	SCE	HEV	PCO	CON
Fornell-larcker Criterion	**SOV**	**0.851**				
	**SCE**	-0.091	**0.839**			
	**HEV**	0.035	-0.128	**0.861**		
	**PCO**	-0.077	0.288	-0.110	**0.900**	
	**CON**	0.381	-0.232	0.647	-0.171	**0.860**
Heterotrait-Monotrait Ratio (HTMT)	**SOV**					
	**SCE**	0.093				
	**HEV**	0.686	0.099			
	**PCO**	0.160	0.536	0.261		
	**CON**	0.651	0.178	0.767	0.253	

*Diagonal elements are the square root of AVE. These values should exceed the Inter-Construct Correlations for adequate discriminant validity.

Note: PCO = Privacy concerns; SCE = self-censorship; SOV = social value; HEV = hedonic value; CON = Continuance intention.

The present study further tested the potential common method bias in our research. First, a Harman’s one-factor test was conducted. The results shown that the five factors were exerted and the largest covariance explained by the single factor was not more than 50%, suggesting that common method bias was not a serious concern in the present study. Second, the present study built a new measurement model that included an additional common method factor and compared it with the original model [24]. The results shown that the variable loading in the original model were all significant at the P<0.001 level, whereas the loadings of the common method factor in the new developed model were not significant. This again indicated that common method bias was unlikely a concern in our study.

### Hypothesis testing

The proposed research model and its corresponding hypotheses were tested by using structure equation modeling (SEM) software LISREL 8.72. Table 4 listed the recommended and actual values of the model fit indices. As shown in the Table, the actual values of the model fit indices were all better than the recommended values, showing a good fitness between our research model and the data [27].

**Table 4:** Fit induces and recommended values (N = 509).

Fit index	*x*^*2*^*/df*	RMSEA	GFI	AGFI	CFI	NFI
Recommended value	<5	<0.08	>0.90	>0.80	>0.90	>0.90
Model value	1.98	0.044	0.961	0.940	0.988	0.976

Notes: RMSEA, root mean square error of approximation; GFI, goodness of fit index; AGFI, adjusted goodness of fit index; CFI, comparative fit index; NFI, normed fit index.

**Fig 2:**
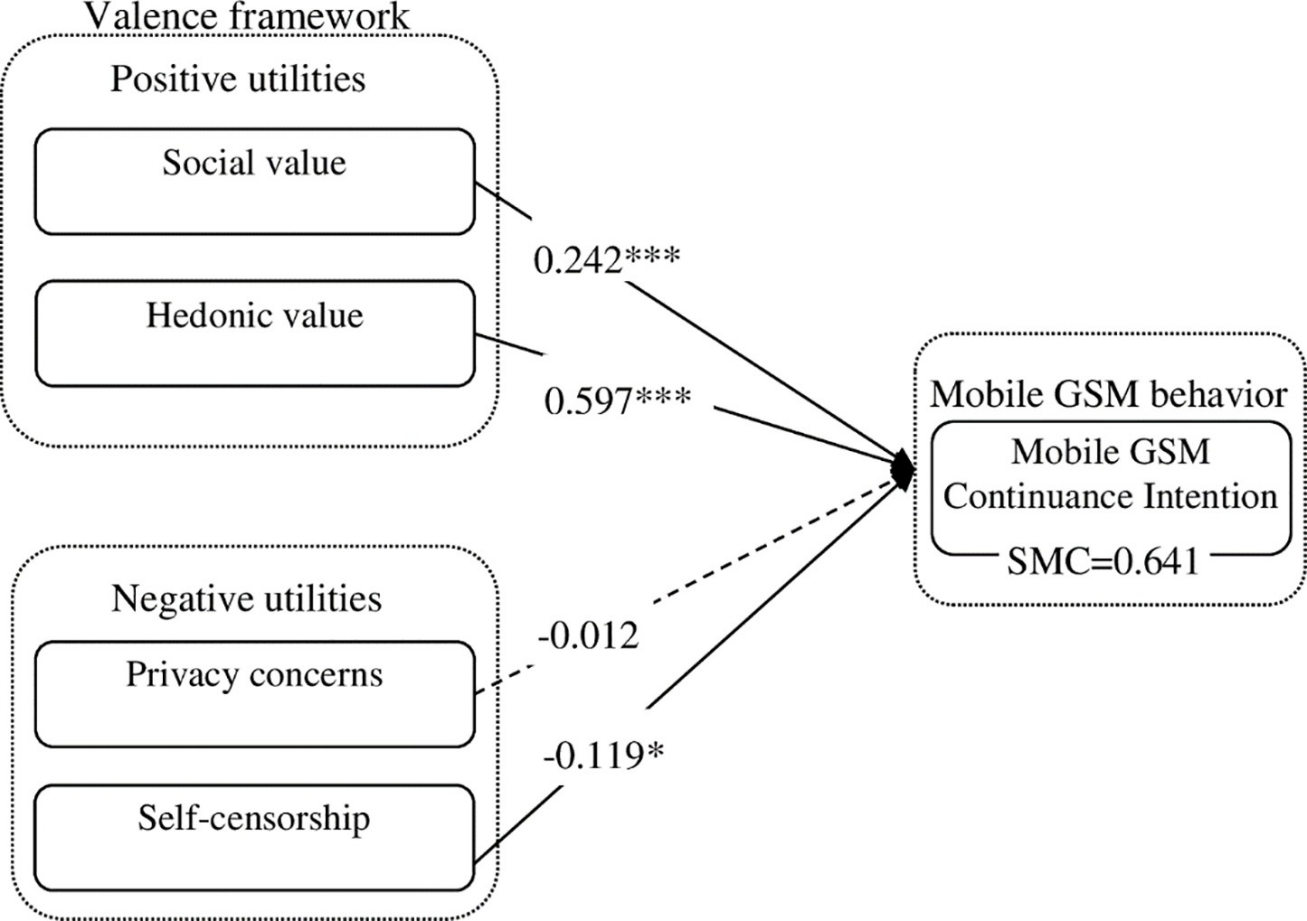
Test results of the research model. * p<0.05; **p<0.01; ***p<0.001.

The results of model testing are displayed in the Fig 2, which shown that most proposed hypotheses are validated by the empirical data. The hypothesized paths from positive utilities factors including social value and hedonic value to mobile GSM continuance were significant at P<0.001, thus validating hypothesis H1 and hypothesis H2. The impact of self-censorship on mobile GSM continuance was negative significant, thus supporting hypothesis H4. However, the influence of privacy concerns on mobile GSM continuance were not significant, thus hypothesis H3 were not supported. The R2 for mobile GSM continuance intention was 0.641, which indicated a reasonable explanation of the variance for the dependent variable.

### Neural network analysis

Following the procedure conducted by extant studies [26, 28, 29], the present study adopted a SEM-NN approach to test our proposed model. Specifically, the multilayer perceptron training algorithm was employed to train the neural network, which was calculated using SPSS 24.0 [30]. The significant predictors calculated by SEM were used as the input layer to build the neural network model. And the output of the neural network is mobile GSM continuance intention.

Following the procedure performed by Zhang, Chen, et al. [30], our study calculates the neural network using one to ten hidden nodes. To predict the accuracy of the NN model, the present study conducted a ten-fold cross-validation analysis [5, 30]. The Root Mean Square Error (RMSE) were calculated to test the prediction accuracy of the NN model. The two models’ average cross-validated RMSE were very small (training data: from 0.170 to 0.176; testing data: from 0.172 to 0.179). This indicated that the neural network model of our study is reliable and accurate prediction [5, 30].

The present study then conducted the sensitivity analyses [28, 29]. The importance of each predictor is listed in Table 5. hedonic value is the strongest factors that affect mobile GSM continuance, following by social value, and self-censorship.

**Table 5:** Sensitivity analysis.

	Output: CON		
	SCE	SOV	HEV
1	0.245	0.344	0.411
2	0.251	0.314	0.435
3	0.259	0.368	0.374
4	0.224	0.400	0.375
5	0.202	0.349	0.415
6	0.245	0.340	0.571
7	0.230	0.291	0.479
8	0.234	0.433	0.333
9	0.203	0.398	0.400
10	0.190	0.374	0.436
Average	0.228	0.361	0.423
Importance	52.777	85.342	100

Note: SCE = self-censorship; SOV = social value; HEV = hedonic value; CON = Continuance intention.

## Discussion

Based on the valence framework, the present study examined citizens’ both positive and negative utilities on their continuance intention to use mobile GSM. The present study reveals several important findings.

Firstly, in terms of positive utilities, the present study found that both social value and hedonic value positively affect mobile GSM continuance intention. This is consistent with the findings of Li, Yang [2] which found that social gratification and enjoyment experience have positive influences on continuance intention to use mobile government social media. Specifically, in terms of path coefficient as well as the significant levels obtained by SEM, hedonic value exerts stronger impact on mobile GSM continuance than that from social value. This is further supported by the results of the neural network model analysis which indicated that hedonic value is more influencing predictor of continuous usage of mobile GSM. This thus highlights the important role of hedonic value (e.g., enjoyment experience and pleasant feeling) in forming citizens’ mobile GSM continuance. It is also worthy to know that citizens’ social value facilitates citizens’ mobile GSM continuance. This suggests that social interaction with citizens also plays an important role in determining mobile GSM continuance.

Secondly, the present study found that self-censorship negatively affects mobile GSM continuance. This is similar to the research of Zhong, Wang [20]. Our findings therefore further provide evidence that the more citizens perceive a high level of self-censorship, the more likely they will discontinue to use mobile GSM. However, the impacts of privacy concerns on mobile GSM continuance were not significant. The plausible explanation is that unlike the traditional mobile financial services (e.g., mobile banking or mobile payment) which involve many personal sensitive information, the usage of mobile government social needs little sensitive information. Therefore, citizens’ concerns about their privacy invasion of using mobile GSM will not significantly affect continuance intention to use mobile GSM.

## Research implications

### Theoretical implications

The present study has several theoretical implications. First of all, many previous studies tend to focus on the technology performance-based enablers to explain mobile social media usage behaviors by extending the leading TAM. In the context of the present study, users of mobile GSM have dual roles included technology user and service consumer. The leading TAM and its extending models unable to explain the underlying mechanism of why and how citizens use mobile GSM to satisfy their various needs. Therefore, the present study applied the valence framework in the mobile GSM context to explain why and how citizens decide to continue using of mobile GSM.

Secondly, unlike the existing studies mainly focused on the benefits aspect to explain mobile social media usage, the present study explored the impact of both positive and negative utilities on citizens’ mobile GSM continuance. Specifically, the positive utilities included hedonic value and social value positively affect citizens’ mobile GSM continuance, while the negative utilities such as self-censorship negatively influence citizens’ mobile GSM continuance. The present study thus contributes to the existing literature by exploring how citizens’ positive and negative utilities might influence their continuance using of mobile GSM.

Finally, unlike the extant studies tend to examine mobile social media usage by adopting SEM approach, our study utilized a SEM-neural network method to explore the factors that affect mobile GSM continuance. The SEM-neural network method can take the advantages of both the theoretical validating-based SEM and non-linear relationships-centered neural network approaches. The present study thus can validate the results by employing multiple methods and find out the most significant factors that determine mobile GSM continuance.

### Practical implications

The present study also has several practical contributions. First, mobile GSM managers should pay close attention to citizens’ positive utilities because our study found that citizens’ social value and hedonic value positively affect their mobile GSM. In terms of hedonic value, mobile GSM managers should provide citizens with pleasure and sense of fun during the process of their mobile GSM usage. For instance, mobile GSM managers can provide personalized responses to citizens based on their preferences and specific contexts by employed big data and artificial intelligence technologies. In terms of citizens’ social value, mobile GSM managers should maintain good relationships with citizens by promptly responding to the comments or suggestions provided by their citizens in mobile GSM platform.

Second, mobile GSM managers should also pay close attention to citizens’ negative utilities [31]. For instance, our study found that self-censorship negative influence their mobile GSM continuance. Mobile GSM managers can reduce citizens’ self-censorship by clearly stating which content or information should not be published online. Such clear explanation will help citizens to reduce their uncertainty perceptions and thus decreasing their self-censorship. For instance, government agencies and mobile GSM managers can classify citizens’ comments and discussions into different topics on the mobile GSM platform to manage them separately and identically. On the other hand, even though, the impact of privacy concerns on mobile GSM continuance is not significant. Mobile GSM managers should also take measures to reduce citizens’ privacy concerns during their usage process.

Finally, government agencies and mobile GSM managers should pay close attention to both positive and negative utilities citizen perceived in the process of their mobile GSM usage. Nowadays, social media regulation has been adopted by many countries and have receiving worldwide attention [2]. Government agencies should take measures to increase citizens’ positive utilities, and decrease their negative utilities. To minimize the negative impact, government agencies and GSM system designers should develop new algorithms based on the big data technology to protect citizens’ privacy. More specifically, the keyword filtering algorithms should be improved to ensure the normal posts and discussion. For instance, the keywork filtering algorithms should include the tendency evaluation based on a particular user’s historical content or logs to minimize the negative influence of self-censorship and perceived privacy.

## Conclusions

Based on the valence framework, the present study examined the impacts of citizens’ positive and negative utilities on their mobile GSM continuance. The results obtained by the SEM-NN approach confirm the usefulness of the valence framework in explaining citizens’ mobile GSM continuance and identify the most significant factors that determine their mobile GSM continuance.

As with all empirical studies, the present study also has a few limitations should be acknowledged. First, it is worth noting that the data in our study were collected from mobile GSM users in a single developing country, e.g., China. Despite focusing on user content in a specific region can reduce unexplained variances obtained from the model calculation, such narrow focus might hinder the generalizability of the results. Therefore, future studies are encouraged to validated our findings by retesting our research model in different countries or regions.

Second, the present study didn’t include actual continuance use in the research model. Despite this limitation is mentioned, it should not undermine the results of our study because the causal relationship between use intention and actual behavior has been validated by extensive empirical studies. However, the existing of different intention measurements might have protentional to affect its predictive power. Future studies thus are encouraged to provide more insight by incorporating the actual continuance usage in the research model.

Finally, to fully capture the antecedents of citizens’ mobile GSM continuance behaviors, an ideal research design would be a longitudinal analysis over a particular different period. However, the cross-sectional nature of our study restricted the longitudinal calculation. Further studies are encouraged to adopt a longitudinal analysis to test mobile GSM continuance.

## Supporting information

S1 Table Scales and items. (PDF)

S2 Table Loadings and cross loading. (PDF)

S1 Data (XLSX)
