# Supplementary material for: Explaining mobile government social media continuance from the valence perspective: A SEM-NN approach
Source: PLoS One. 2021 Feb 4;16(2):e0246483. doi: 10.1371/journal.pone.0246483 (PMC7861361; doi:10.1371/journal.pone.0246483)
Supplement: S1 Table — (PDF) [file pone.0246483.s001.pdf]

S1 Table. Scales and items

Privacy concern [16]
PCO1: I am concerned that information I submit on the government microblogging APP could be misused.
PCO2: I am concerned because information I transmit on the government microblogging APP can be intercepted by third parties.
PCO3: I am concerned about submitting personal information on the government microblogging APP because it could be used in a way I did not foresee.
Social value [23, 24]
SOV1: I use the government microblogging App to share ideas with other citizens
SOV2: Sending information to other citizens using the government microblogging App can improve my image.
SOV3: Sharing information with other citizens using the government microblogging App can improve relationship.
Hedonic Value [23]
HEV1: I have fun interacting with the government microblogging APP.
HEV2: Using the government microblogging APP provides me with a lot of enjoyment.
HEV3: I enjoy using the government microblogging APP.
Self-censorship [22]
When using the government microblogging App:
SCE1：I usually delete a status update before posting.
SCE2：I often change the wording of a status update to avoid angering some of my microblogging friends
SCE3：I often delete a status update I am already posted.
Continuance Intention [25]
CON1: If could, I will continue using the government microblogging APP.
CON2: I will continue using the government microblogging APP in the future.
CON3: I will recommend my friends and family members to use the government microblogging APP.
