# Supplementary material for: Explaining mobile government social media continuance from the valence perspective: A SEM-NN approach
Source: PLoS One. 2021 Feb 4;16(2):e0246483. doi: 10.1371/journal.pone.0246483 (PMC7861361; doi:10.1371/journal.pone.0246483)
Supplement: S2 Table — (PDF) [file pone.0246483.s002.pdf]

S2 Table. Loadings and cross loading

Factor	PCO	CON	SOV	SCE	HEV
PCO1	**.813**	.057	-.043	.174	-.153
PCO2	**.897**	-.114	-.071	.201	-.047
PCO3	**.870**	-.164	-.024	.225	-.028
CON1	-.115	**.778**	.176	-.134	.238
CON2	-.093	**.794**	.187	-.027	.279
CON3	-.003	**.776**	.266	-.023	.211
SOV1	-.013	.088	**.761**	-.014	.337
SOV2	-.053	.245	**.831**	.025	.176
SOV3	-.069	.317	**.783**	.063	.116
SCE1	.162	-.063	-.095	**.806**	.008
SCE2	.206	-.024	.133	**.786**	.024
SCE3	.186	-.062	.026	**.827**	-.062
HEV1	-.020	.313	.222	-.034	**.788**
HEV2	-.113	.204	.213	.000	**.828**
HEV3	-.143	.409	.261	.009	**.653**

Note: Bold is the internal-construct loading of each extracted factor. PCO=Privacy concerns; SCE=self-censorship; SOV=social value; HEV=hedonic value; CON=Continuance intention
